# Association of Molnupiravir and Nirmatrelvir-Ritonavir with reduced mortality and sepsis in hospitalized omicron patients: a territory-wide study

**DOI:** 10.1038/s41598-023-35068-w

**Published:** 2023-05-15

**Authors:** Abraham Ka-chung Wai, Teddy Tai-loy Lee, Sunny Ching-long Chan, Crystal Ying Chan, Edmond Tsz-fung Yip, Luke Yik-fung Luk, Joshua Wing-kei Ho, Kevin Wang-leong So, Omar Wai-kiu Tsui, Man-lok Lam, Shi-yeow Lee, Tafu Yamamoto, Chak-kwan Tong, Man-sing Wong, Eliza Lai-yi Wong, Timothy Hudson Rainer

**Affiliations:** 1 Department of Emergency Medicine, School of Clinical Medicine, LKS Faculty of Medicine, The University of Hong Kong, Room 101, 1/F, University of Hong Kong the Hong Kong Jockey Club Building for Interdisciplinary Research, 5 Sassoon Road, Pokfulam, Hong Kong SAR, China; 2 Accident and Emergency, Queen Mary Hospital, Hong Kong SAR, China; 3 Accident and Emergency, The University of Hong Kong – Shenzhen Hospital, Shenzhen, China; 4 Centre for Health Systems & Policy Research, JC School of Public Care and Primary Care, Faculty of Medicine, The Chinese University of Hong Kong, Hong Kong SAR, China; 5 Laboratory of Data Discovery for Health, Hong Kong Science and Technology Park, Hong Kong SAR, China; 6 Accident and Emergency, Yan Chai Hospital, Hong Kong SAR, China; 7 Department of Medicine and Geriatric, Princess Margaret Hospital, Hong Kong SAR, China; 8 Department of Land Surveying and Geo-Informatics, The Hong Kong Polytechnic University, Hong Kong SAR, China

**Keywords:** Outcomes research, Epidemiology, Infectious diseases, Respiratory tract diseases

## Abstract

This study evaluates the association between antivirals (Molnupiravir and Nirmatrelvir-Ritonavir) and all-cause and respiratory mortality and organ dysfunction among high-risk COVID-19 patients during an Omicron outbreak. Two cohorts, Nirmatrelvir-Ritonavir versus control and Molnupiravir versus control, were constructed with inverse probability treatment weighting to balance baseline characteristics. Cox proportional hazards models evaluated the association of their use with all-cause mortality, respiratory mortality, and all-cause sepsis (a composite of circulatory shock, respiratory failure, acute liver injury, coagulopathy, and acute liver impairment). Patients recruited were hospitalized and diagnosed with the COVID-19 Omicron variant between February 22, 2022 and April 15, 2022, and followed up until May 15, 2022. The study included 17,704 patients. There were 4.67 and 22.7 total mortalities per 1000 person-days in the Nirmatrelvir-Ritonavir and control groups respectively before adjustment (weighted incidence rate ratio, − 18.1 [95% CI − 23.0 to − 13.2]; hazard ratio, 0.18 [95% CI, 0.11–0.29]). There were 6.64 and 25.9 total mortalities per 1000 person-days in the Molnupiravir and control groups respectively before adjustment (weighted incidence rate ratio per 1000 person-days, − 19.3 [95% CI − 22.6 to − 15.9]; hazard ratio, 0.23 [95% CI 0.18–0.30]). In all-cause sepsis, there were 13.7 and 35.4 organ dysfunction events per 1000 person-days in the Nirmatrelvir-Ritonavir and control groups respectively before adjustment (weighted incidence rate ratio per 1000 person-days, − 21.7 [95% CI − 26.3 to − 17.1]; hazard ratio, 0.44 [95% CI 0.38–0.52]). There were 23.7 and 40.8 organ dysfunction events in the Molnupiravir and control groups respectively before adjustment (weighted incidence ratio per 1000 person-days, − 17.1 [95% CI, − 20.6 to − 13.6]; hazard ratio, 0.63 [95% CI 0.58–0.69]). Among COVID-19 hospitalized patients, use of either Nirmatrelvir-Ritonavir or Molnupiravir compared with no antiviral use was associated with a significantly lower incidence of 28-days all-cause and respiratory mortality and sepsis.

## Introduction

Since the first outbreak of COVID-19 at the end of 2019, SARS-CoV-2 has led to a pandemic resulting in 530 million confirmed cases and 6.3 million mortalities in the past 30 months^1^. Health authorities in different countries attempted to prevent and control the disease by population-wide, non-pharmaceutical interventions, vaccination programs, and the introduction of novel antiviral and immune-modulating agents. Several variants have been identified since then; notably, the Omicron variant of SARS-CoV-2 was declared a variant of concern (VOC) in late November 2021^2^. It is associated with more efficient cell entry, immune evasion, and increased infectivity^3^.

Patients infected with the Omicron variant may demonstrate a full spectrum of disease^4^. The manifestation of pathology may be found in the respiratory^5^, cardiovascular^6^, hepatic^4^, renal^7^, coagulation^8^ and nervous systems^9^, in addition to mortality. In this connection, these patients are at risk of developing sepsis^10,11^ as defined by Sepsis-3 criteria^12^. Viral sepsis is highly prevalent among hospitalized COVID-19 patients, with a reported 77.9% incidence in patients admitted into intensive care units and 33.3% admitted in general wards^13^. Organ dysfunction resulting from sepsis, such as circulatory shock, respiratory failure, acute kidney and liver injuries and coagulopathies may increase risk of death in COVID-19 patients. The early administration of appropriate antivirals in suspected or confirmed respiratory viral infection may stop viral replication, reduce viral load and prevent and manage severe infection^14^.

The development of effective oral antivirals against SARS-CoV-2 gives hope to clinicians to manage COVID-19 patients with high risk to sepsis, including neonates and young children, pregnant women, older adults, and immunosuppressed individuals^15^, especially when they do not complete COVID-19 vaccination in the community^16^. Molnupiravir, an inhibitor of the viral RNA-dependent RNA polymerase (RdRp), and Nirmatrelvir-Ritonavir, an irreversible inhibitor of SARS-CoV-2 main protease (Mpro), are effective against the Omicron variant^17^.

After the first isolation of the Omicron variant in Hong Kong in January 2022, the health systems in Hong Kong were overwhelmed during mid-February to mid-April 2022 (5th wave), leading to 1.18 million reported cases and 8,735 mortalities. At the peak of the 5th wave, only 28% of the population received the booster dose^18^, which is necessary for substantial protection against the Omicron variant^19^, and which is regarded a major contributor to the high mortality. Seventy-one percent (71%) of mortalities were contributed by patients aged 80 years or above, among which 74.5% were unvaccinated and only 0.5% received the booster dose. The oral antivirals have potential to confer protection from developing severe infection to these patients who have not completed their vaccination or booster.

The effectiveness of these antivirals against the Omicron variant to prevent mortality and organ dysfunction among high-risk hospitalized patients is unclear. This study evaluates the association between antivirals (Molnupiravir and Nirmatrelvir-Ritonavir) and all-cause and respiratory mortality and organ dysfunction among high-risk hospitalized COVID-19 patients during an Omicron outbreak.

## Methods

### Study design and setting

All patients with COVID-19 in Hong Kong were identified through the Clinical Data Analysis and Reporting System (CDARS), which is a territory-wide administrative database managed by the Hospital Authority (HA), the statutory body operating 42 public hospitals and 120 clinics for the population sized 7.4 million in Hong Kong^20^. This database collects anonymized demographic, clinical and service data, and has been used extensively for large population studies^21,22^. The Hospital Authority Hong Kong West Cluster/The University of Hong Kong institutional review board (UW 22-258) approved and waived the requirement for obtaining patient informed consent, and all methods were performed in accordance with relevant guidelines and regulations.

### Selection of participants

Patients who fulfilled the following inclusion criteria were included: (1) a diagnosis of COVID-19 under the International Classification of Diseases, Ninth Revision, Clinical Modified (ICD-9-CM); (2) admitted to hospitals under the Hospital Authority during February 22 to April 15, 2022. We defined the index date as the date of admission of an episode coded for COVID-19.

### Interventions

Our exposure of interest was Molnupiravir and Nirmatrelvir-Ritonavir among hospitalized COVID-19 patients, with an increased risk of deterioration, including old-age and chronic disease patients. We included hospitalized patients who were aged ≥ 60 years; or younger patients aged ≥ 18 with at least one chronic disease. Patients with prescription records of Molnupiravir or Nirmatrelvir-Ritonavir within 4 days of hospital admission were allocated into the Molnupiravir and Nirmatrelvir-Ritonavir treatment groups; patients who received both antivirals were excluded from this study. Considering the delayed access to antivirals in early stage of rolling out, patients received Molnupiravir or Nirmatrelvir-Ritonavir later than 4 days from the index date were excluded.

### Outcomes

The primary outcomes were 28-days all-cause mortality and respiratory mortality, as detailed in Supplementary Table 1. Secondary outcomes were circulatory shock, respiratory failure, acute kidney injury, coagulopathy, acute liver impairment, and combined organ dysfunction, a composite outcome defined as presence of any one of these other secondary outcomes. Acute organ dysfunction is defined by vasopressor initiation, mechanical ventilation, elevation of lactate, or changes in total bilirubin, platelets or creatinine relative to specified baseline values^23^, and the unprecedented prescription of dexamethasone, as directed by the clinical guideline^24^. We defined these outcomes in reference to a sepsis surveillance toolkit, published by the US Centers for Disease Control and Prevention (CDC), which is a validated approach based on administrative data to facilitate health care facilities to monitor the incidence and outcomes of patients who developed sepsis according to the Sepsis-3 criteria, known as an Adult Sepsis Event (ASE)^25^. ASE is defined as (1) presumed serious infection, signified by obtained microbiological examination (for example, blood culture) and ≥ 4 consecutive days of antimicrobials (or up until 1 day before mortality, discharge to hospice, transfer to another acute care hospital, or transition to comfort measures) starting within 2 calendar days of when blood cultures were obtained, plus (2) evidence of concurrent organ dysfunction, signified by any of 6 binary indicators of cardiovascular, pulmonary, renal, hepatic, coagulation, or perfusion dysfunction. This approach for the identification of sepsis patients in Hong Kong data from CDARS has been validated, with high sensitivity (0.93), high specificity (0.86) and area under receiver operating characteristic curve (0.90)^26^.

The follow-up of each patient was commenced from the index date, defined as the date of hospital admission, for 28 days or until date of mortality. For secondary outcomes, follow-up time was defined as time from the index date to time of event, or loss of follow up.

### Covariates

Baseline data was collected for each patient including sex, age, and socioeconomic status. The socioeconomic status of each patient was determined by the Social Deprivation Index (SDI) of their area of residence^27^. SDI is derived from six variables to describe the conditions of social deprivation for each residential district: the proportions of the population with unemployment, monthly household income < US$250, no schooling at all, one-person household, never-married status, and subtenancy. The data comes from population census published by the government. Each of these six variables had significant factor loading for a specific principal factor, and all of them are deemed to be representative indicators of social disadvantage in the published literature and in the setting of the Hong Kong population. SDI for each district was calculated by taking the average of these six selected variables^28^.

Baseline data was also collected for comorbidities (Charlson Comorbidity Index (CCI), diabetes mellitus, hypertension, stroke, congestive heart failure, atrial fibrillation, schizophrenia, cirrhosis, depression, chronic kidney disease, rheumatoid arthritis, obesity and alcohol abuse) and chronic medication use (antiplatelets and anticoagulants, ACE inhibitors and angiotensin receptor blockers, beta blockers, calcium channel blockers, diuretics, statins systemic corticosteroids, bronchodilators and inhaled corticosteroids, cancer drugs, and rheumatological drugs). The differences in patient characteristics between treatment groups were expressed in terms of standardized mean differences (SMD), where covariates with SMD < 0.2 were considered balanced. The International Classification of Diseases, Ninth Revision (ICD-9) codes used for classification of the comorbidities are available in Supplementary Table 2.

### Analysis

Baseline characteristics were expressed as the mean (Standard deviation [SD]) for continuous variables and frequency (%) for categorical variables. Inverse probability treatment weighting (IPTW) based on propensity scores using the aforementioned variables was used to construct a weighted cohort of patients to address potential indication bias due to nonrandomized allocation of patients to the treatment group. IPTW balances baseline characteristics in comparator groups by weighting each patient by the inverse probability of receiving the treatment. Hazard ratios (HR) for the association of Nirmatrelvir-Ritonavir or Molnupiravir use and the study outcomes were estimated over the entire follow-up with weighted Cox proportional hazards regression using weights obtained by IPTW.

Alternatively, given the small event rate, we repeated all analyses utilizing bootstrapping methods followed by sampling with replacement to obtain precise estimates of HRs, incidence rate differences (IRD) and their 95% confidence intervals. Details regarding the bootstrapping method are documented in Supplementary Fig. 1.

Prespecified subgroup analyses were conducted by stratifying the study population by age groups (5-years increments from < 60 to ≥ 80), sex, SDI, and presence of diabetes. Further analyses were conducted to investigate the number of mortalities stratified by individual components of organ dysfunction. The number of organ dysfunctions and length of stay were also stratified by survival status at the end of follow-up.

All significance tests were two-tailed and considered significant when the *P* value was less than 0.05. All analyses were conducted using RStudio version 1.4.1717^29^, using the following packages: ***tidymodels*** was used to obtain bootstrapped dataframes^30^, for the subsequent computation of hazard ratios using the ***survival*** package^31^, and weighted incidence rate differences via the ***fmsb*** package^32,33^. The package ‘WeightIt’ was used to compute the propensity scores and inverse probability weights^32^. The package ***comorbidity*** was used to compute the CCI^34^.

### Ethics approval and consent to participate

This study was approved, and the requirement for obtaining patient informed consent was waived, by the Hospital Authority Hong Kong West Cluster/The University of Hong Kong institutional review board (UW 22-258).

## Results

### Characteristics of study subjects

From February 22, 2022 to April 15, 2022, 17,925 patients were hospitalized with COVID-19. Of these, 44 patients were excluded for missing demographic data. Nirmatrelvir-Ritonavir was prescribed to 770 patients of whom 105 were excluded either because of the mixed use of antivirals (N = 84) or initiating Nirmatrelvir-Ritonavir beyond 4 days after hospital admission (N = 21), leaving 665 for further analysis. Of 1784 given Molnupiravir, 156 were excluded due to mixed use of antivirals (N = 84) or initiating Molnupiravir beyond 4 days after hospital admission (N = 72), leaving 1628 for analysis. These patients with antivirals were weighted against 15,411 patients without antiviral (control) to generate two study cohorts. Supplementary Fig. 2 documents the selection process for the two study cohorts.

Tables 1 and 2 shows patient demographics of patients receiving each antiviral weighted against control, before and after propensity score weighting. Of 665 patients with Nirmatrelvir-Ritonavir use, the mean age was 76.8 (SD, 14) years, 49.9% were men, and 76.1% had a CCI of 0. Among 1,628 with Molnupiravir, the mean age was 80.0 (SD, 13) years, 51.9% were men, and 70.9% had a CCI of 0. In the control group, the mean age was 72.2 years (SD, 25), 54.5% were men, and 71.9% had a CCI of 0.

**Table 1:** Demographics of 16,076 Hospitalized COVID-19 Patients, Before and After Inverse Probability Treatment Weighting.

Variable	Crude			*P* value	After inverse probability treatment weighting			*P* value
	Nirmatrelvir-ritonavir (n = 665)	Control (n = 15,411)	Standardized difference		Nirmatrelvir-ritonavir (n = 665.6)	Control (n = 665.0)	Standardized difference	
Age, mean(SD), y	76.83 (14.00)	72.21 (25.30)	0.23	< 0.001	76.83 (14.00)	73.89 (23.17)	0.15	< 0.001
< = 65	17.1	22.9	0.20	< 0.001	17.1	17.1	< 0.01	0.925
66–75	22.0	15.3			22.0	22.1		
> 75	60.9	61.8			60.9	60.8		
Sex, (%)			0.09	0.593			< 0.01	0.965
Men	49.9	54.5			49.9	49.9		
Women	50.1	45.5			50.1	50.1		
Social Deprivation Index, (%)			0.28	< 0.001			< 0.01	0.999
Q1	19.8	13.2			19.8	20.0		
Q2	29.3	36.5			29.3	29.3		
Q3	39.5	33.2			39.5	39.4		
Q4	11.3	17.1			11.3	11.3		
Comorbidities, (%)								
Charlson comorbidity index			0.10	< 0.001			< 0.01	0.845
0	76.1	71.9			76.1	76.0		
1–2	23.6	27.7			23.6	23.7		
3–4	0.3	0.4			0.3	0.3		
5 +	0.0	0.0			0.0	0.0		
Diabetes	6.8	6.6	0.01	0.548	6.8	6.8	< 0.01	0.954
Hypertension	7.7	7.4	0.01	0.097	7.7	7.7	< 0.01	0.906
Stroke	0.9	1.1	0.02	0.043	0.9	0.9	< 0.01	0.910
Congestive heart failure	2.1	2.6	0.03	< 0.001	2.1	2.1	< 0.01	0.879
Atrial fibrillation	2.4	3.3	0.05	0.012	2.4	2.4	< 0.01	0.932
Schizophrenia	0.6	0.4	0.02	0.990	0.6	0.6	< 0.01	0.980
Liver cirrhosis	0.9	0.2	0.09	0.412	0.9	1.0	< 0.01	0.659
Depression	0.3	0.2	0.01	0.861	0.3	0.3	< 0.01	0.954
Chronic kidney disease	2.7	8.9	0.27	< 0.001	2.7	2.7	< 0.01	0.961
Rheumatoid arthritis	0.3	0.4	0.01	1.000	0.3	0.3	< 0.01	0.879
Obesity	1.1	0.5	0.06	0.861	1.1	1.1	< 0.01	0.797
Alcohol abuse	0.0	0.1	0.05	1.000	0.0	0.0	< 0.01	0.857
Medication use, (%)								
Antiplatelets and anticoagulants	24.4	30.1	0.13	< 0.001	24.4	24.3	< 0.01	0.777
ACE inhibitors and Angiotensin receptor blockers	27.8	27.1	0.02	< 0.001	27.8	27.9	< 0.01	0.652
Beta blockers	17.9	19.4	0.04	< 0.001	17.9	17.8	< 0.01	0.803
Calcium channel blockers	37.1	38.0	0.02	< 0.001	37.1	37.2	< 0.01	0.723
Diuretics	11.0	16.8	0.17	< 0.001	11.0	10.9	< 0.01	0.877
Statins	31.4	33.1	0.04	< 0.001	31.4	31.5	< 0.01	0.679
Systemic corticosteroids	3.3	5.2	0.10	< 0.001	3.3	3.3	< 0.01	0.877
Antidiabetics	20.5	20.0	0.01	< 0.001	20.5	20.5	< 0.01	0.657
Bronchodilators and Inhaled corticosteroids	0.6	0.8	0.02	0.033	0.6	0.6	< 0.01	0.997
Cancer drugs	0.3	0.2	0.01	0.548	0.3	0.3	< 0.01	0.962
Rheumatological drugs	5.1	7.5	0.10	< 0.001	5.1	5.1	< 0.01	0.879

**Table 2:** Demographics of 17,039 Hospitalized COVID-19 Patients, Before and After Inverse Probability Treatment Weighting.

Variable	Crude			*P* value	After inverse probability treatment weighting			*P* value
	Molnupiravir (n = 1628)	Control (n = 15,411)	Standardized difference		Molnupiravir (n = 1628.0)	Control (n = 1628.6)	Standardized difference	
Age, mean(SD), y	80.01 (13.21)	72.21 (25.30)	0.39	< 0.001	80.01 (13.21)	77.52 (20.04)	0.15	< 0.001
< = 65	12.5	22.9	0.28	< 0.001	12.5	12.5	< 0.01	0.999
64–75	17.5	15.3			17.5	17.5		
≥ 75	70.0	61.8			70.0	70.0		
Sex, (%)			0.05	0.252			< 0.01	0.974
Men	51.9	54.5			51.9	51.9		
Women	48.1	45.5			48.1	48.1		
Social Deprivation Index, No. (%)			0.12	< 0.001			< 0.01	1.000
Q1	17.4	13.2			17.4	17.3		
Q2	34.8	36.5			34.8	34.9		
Q3	32.2	33.2			32.2	32.2		
Q4	15.6	17.1			15.6	15.6		
Comorbidities, (%)								
Charlson Comorbidity Index			0.03	0.274			< 0.01	0.993
0	70.9	71.9			70.9	71.0		
1–2	28.7	27.7			28.7	28.8		
3–4	0.4	0.4			0.4	0.4		
5 +	0.0	0.0			0.0	0.0		
Diabetes	7.4	6.6	0.03	0.089	7.4	7.4	< 0.01	0.938
Hypertension	9.7	7.4	0.08	< 0.001	9.7	9.8	< 0.01	0.939
Stroke	0.7	1.1	0.04	0.283	0.7	0.7	< 0.01	0.994
Congestive heart failure	2.0	2.6	0.04	0.047	2.0	2.1	< 0.01	0.992
Atrial fibrillation	3.1	3.3	0.01	0.554	3.1	3.1	< 0.01	0.995
Schizophrenia	0.6	0.4	0.02	0.204	0.6	0.6	< 0.01	0.897
Liver cirrhosis	0.1	0.2	0.04	1.000	0.1	0.1	< 0.01	0.980
Depression	0.3	0.2	0.01	0.879	0.3	0.3	< 0.01	0.977
Chronic kidney disease	10.7	8.9	0.06	< 0.001	10.7	10.7	< 0.01	0.893
Rheumatoid arthritis	9.5	0.4	0.01	0.265	0.4	0.4	< 0.01	0.973
Obesity	0.5	0.5	< 0.01	1.000	0.5	0.5	< 0.01	0.909
Alcohol abuse	0.1	0.1	0.02	1.000	0.1	0.1	< 0.01	0.931
Medication use, (%)								
Antiplatelets and anticoagulants	37.5	30.1	0.16	0.117	37.5	37.6	< 0.01	0.914
ACE inhibitors and Angiotensin receptor blockers	34.0	27.1	0.15	0.108	34.0	34.0	< 0.01	0.895
Beta blockers	24.8	19.4	0.13	0.117	24.8	24.8	< 0.01	0.914
Calcium channel blockers	45.2	38.0	0.15	0.836	45.2	45.2	< 0.01	0.914
Diuretics	20.2	16.8	0.09	0.656	20.2	20.2	< 0.01	0.981
Statins	41.2	33.1	0.17	0.099	41.2	41.3	< 0.01	0.908
Systemic corticosteroids	6.6	5.2	0.06	0.519	6.6	6.6	< 0.01	0.893
Antidiabetics	24.9	20.0	0.12	0.240	24.9	25.0	< 0.01	0.893
Bronchodilators and Inhaled corticosteroids	9.5	0.8	0.06	0.019	0.3	0.3	< 0.01	0.992
Cancer drugs	0.6	0.2	0.05	0.103	0.6	0.6	< 0.01	0.884
Rheumatological drugs	9.5	7.5	0.07	0.461	9.5	9.4	< 0.01	0.948

*ACE* Angiotensin converting enzyme.

Before weighting, Nirmatrelvir-ritonavir users had a lower incidence of chronic kidney disease (2.7%) compared to control (8.9%); there was also less use of diuretics in Nirmatrelvir-ritonavir users (11.0%) compared to control (16.8%). Molnupiravir users were more senile, with more patients aged ≥ 75 years (70.0%) compared to control (61.8%). Compared to control, molnupiravir users used more medications, including antiplatelets and anticoagulants (37.5% vs. 30.1%), ACE inhibitors and angiotensin receptor blockers (34.0% vs. 27.1%), beta blockers (24.8% vs. 19.4%), calcium channel blockers (45.2% vs. 38.0%), statins (41.2% vs. 33.1%), and antidiabetic medications (24.9% vs. 20.0%). These differences were well addressed through IPTW where all weighted variables had a SMD < 0.01 after IPTW (Tables 1 and 2).

### Main results: primary outcomes

**Figure 1:**
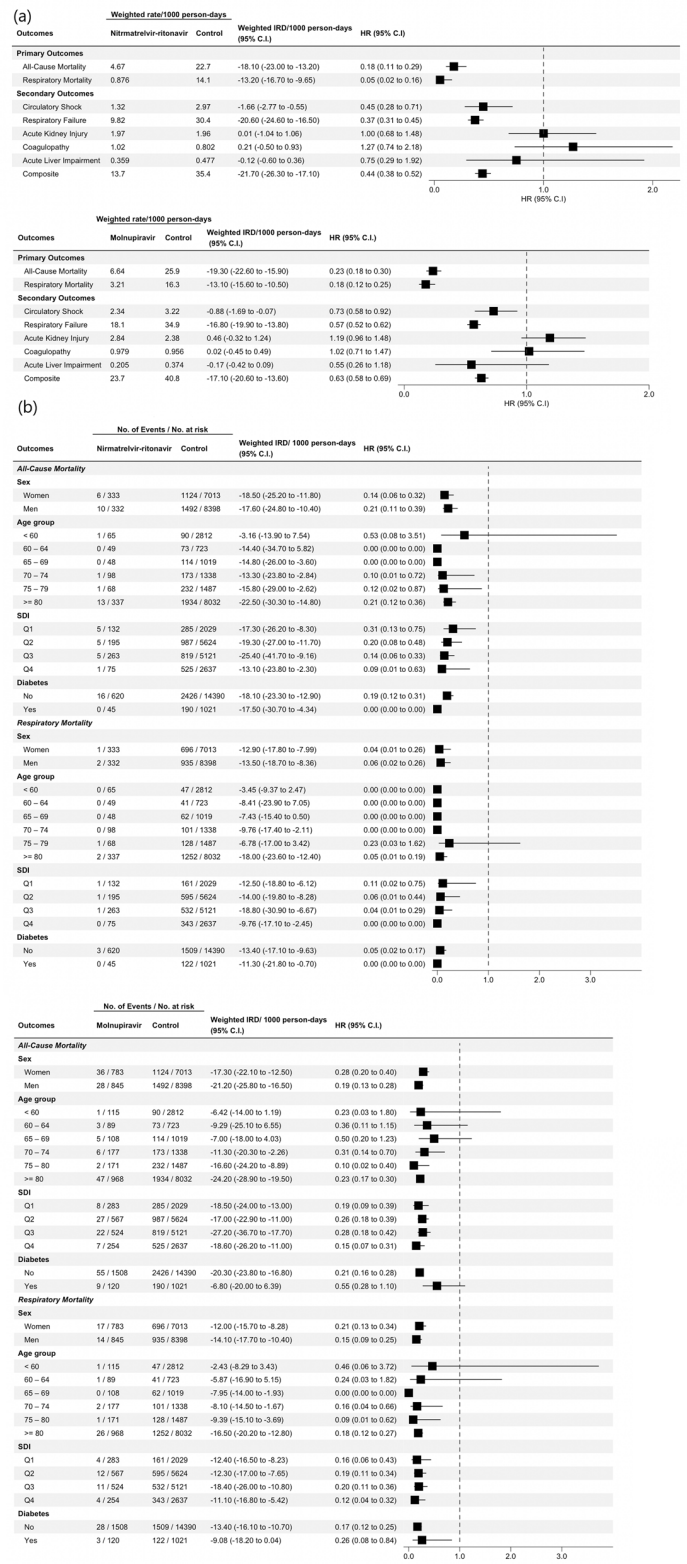
Association of Antivirals Use with Mortality and Organ Dysfunction. (**a**) Association between Nirmatrelvir-Ritonavir and Molnupiravir Use, All-Cause Mortality, and Major Organ Dysfunction Events in 17.704 Hospitalized Patients Infected with Omicron Variant of SARS-CoV-2; (**b**) Association between Nirmatrelvir-Ritonavir and Molnupiravir Use, All-Cause and Respiratory Mortality, Stratified by Age, Sex, SDI and Diabetes. Abbreviations: *IRD* incidence rate difference; *HR* hazard ratio; *SDI* social deprivation index. Weighted incidence rate differences comparing Nirmatrelvir-Ritonavir and Molnupiravir Users to non-users after inverse probability weighting was applied. Composite: Time to first circulatory shock, respiratory failure, coagulopathy, acute kidney injury and acute liver impairment. There were fewer composite events compared with total individual events, as participants were censored at first event of interest.

Nirmatrelvir-Ritonavir use was significantly associated with a lower risk of all-cause mortality (HR 0.18 [95% CI 0.11–0.29]) and respiratory mortality (Hazard ratio [HR], 0.05 [95% CI 0.02–0.16]) (Fig. 1a). Molnupiravir was similarly significantly associated with a lower risk of all-cause mortality (HR 0.23 [95% CI 0.18–0.30]) and respiratory mortality (HR 0.18 [95% CI 0.12–0.25]) (Fig. 1a).

In subgroup analysis of the Nirmatrelvir-Ritonavir / control cohort, the HRs of all-cause mortality among patients aged 70–74, 75–79, and ≥ 80 years were 0.10 (0.01–0.72), 0.12 (0.02–0.87), and 0.21 (0.12–0.36). There were no events among patients aged 60–64 and 65–69 years. The risk decreased with different SDI groups, where the HRs of Q1 (best socioeconomic status), Q2, Q3, and Q4 (worst) were 0.31 (0.13–0.75), 0.20 (0.08–0.48), 0.14 (0.06–0.33), and 0.09 (0.01–0.63) respectively, with a similar pattern for respiratory mortality (Fig. 1b).

In the Molnupiravir/control cohort, the HRs of all-cause mortality among patients aged 60–64, 65–69, 70–74, 75–79, and ≥ 80 years were 0.36 (0.11–1.15), 0.50 (0.20–1.23), 0.31 (0.14–0.70), 0.10 (0.02–0.40), and 0.23 (0.17–0.30). All SDI groups were associated with decreased risks of call-cause mortality, where the HRs of Q1, Q2, Q3, and Q4 were 0.19 (0.09–0.39), 0.26 (0.18–0.39), 0.28 (0.18–0.42), and 0.15 (0.07–0.31) respectively; and respiratory mortality 0.16 (0.06–0.43), 0.19 (0.11–0.34), 0.20 (0.11–0.36) and 0.12 (0.04–0.32) respectively (Fig. 1b).

### Secondary outcomes

In the Nirmatrelvir-Ritonavir/control cohort, 1840 patients experienced the composite endpoint of organ dysfunction, including 952 with circulatory shock, 130 with respiratory failure, 300 with coagulopathy, 703 with acute kidney injury (AKI), and 125 with acute liver impairment. There were 13.7 and 35.4 composite events per 1000 person-days (weighted IRD/1000 person-days, − 21.7 [95% CI − 26.3 to − 17.1]) for user vs nonuser. After adjustment, the HR for the composite organ dysfunction outcome was 0.44 (0.38–0.52). Corresponding HRs were 0.45 (0.28–0.71) for circulatory shock and 0.37 (0.31–0.45) for respiratory failure. A statistically significant HR was not observed for AKI (HR 1.00 [95% CI 0.68–1.48]), coagulopathy (HR 1.27 [95% CI 0.74–2.18]), and acute liver impairment (HR, 0.75 [95% CI 0.29–1.92]). (Fig. 1a).

In the Molnupiravir/control cohort, 1979 patients experienced the composite outcome, with a total of 1011 with circulatory shock, 131 with respiratory failure, 319 with coagulopathy, 770 with AKI, and 127 with acute liver impairment. There were 23.7 and 40.8 composite events per 1000 person-days (weighted IRD/1000 person-days, − 17.1 [95% CI − 20.6 to − 13.6]) for user vs nonuser. After adjustment, a statistically significant HR was only observed for the composite outcome (HR 0.63 [95% CI 0.58–0.69]), circulatory shock (HR 0.73 [95% CI 0.58–0.92]), and respiratory failure (HR 0.57 [95% CI 0.52–0.62]). The risk of coagulopathy (HR 1.03 [95% CI 0.71–1.47]), acute liver impairment (HR 0.55 [95% CI 0.26–1.18]), and AKI (HR 1.19 [95% CI 0.96–1.48]) were not significant (Fig. 1a).

**Figure 2:**
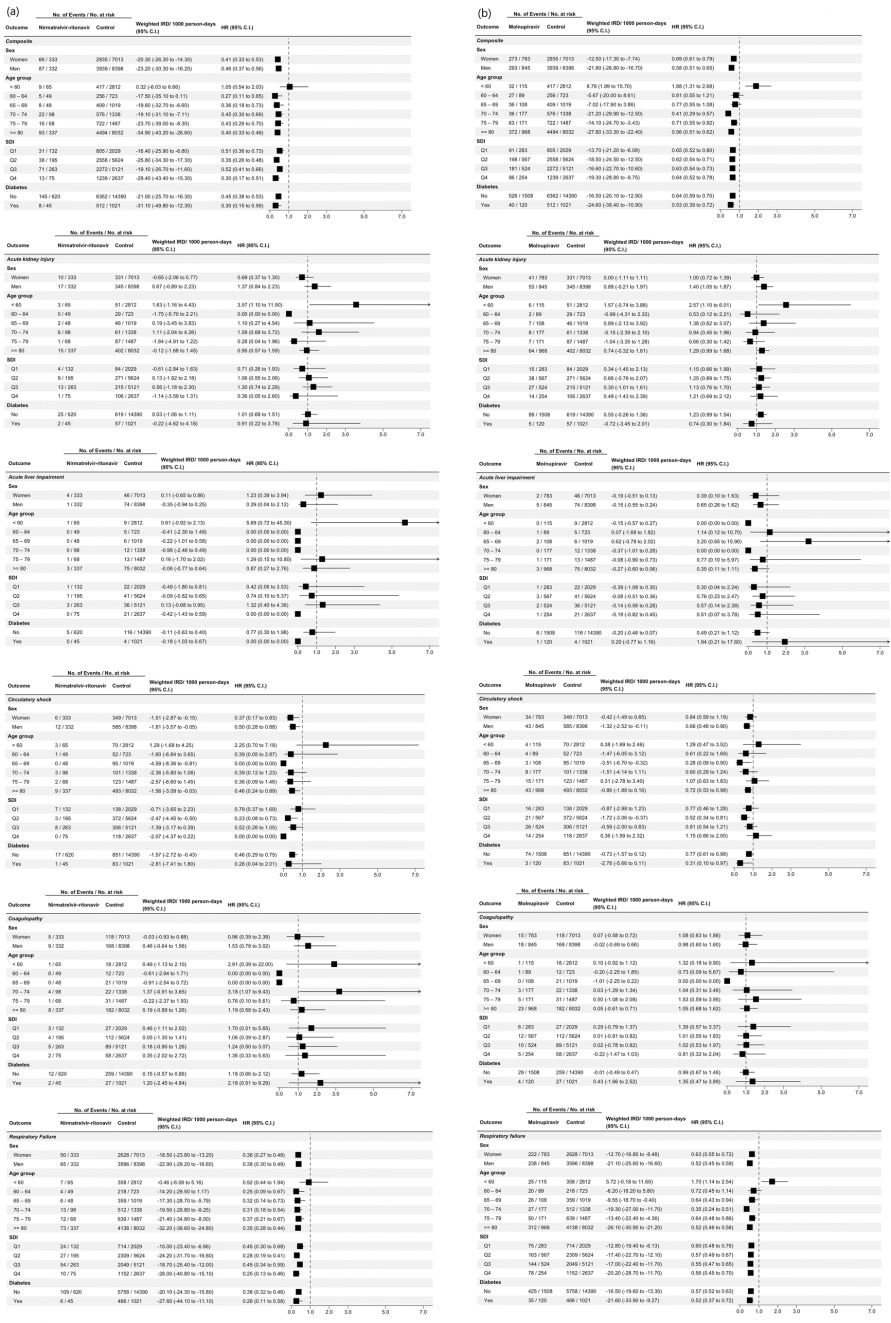
Association of Antivirals Use with Organ Dysfunction, Stratified by Age, Sex, SDI and Diabetes. (**a**) Association between Nirmatrelvir-Ritonavir and Major Organ Dysfunction Events in Hospitalized Patients Infected with Omicron Variant of SARS-CoV-2; (**b**) Association between Molnupiravir and Major Organ Dysfunction Events in Hospitalized Patients Infected with Omicron Variant of SARS-CoV-2. Abbreviations: *IRD* incidence rate difference; *HR* hazard ratio; *SDI* social deprivation index. Weighted incidence rate differences comparing Nirmatrelvir-Ritonavir and Molnupiravir Users to non-users after inverse probability weighting was applied. Composite: Time to first circulatory shock, respiratory failure, coagulopathy, acute kidney injury and acute liver impairment. There were fewer composite events compared with total individual events, as participants were censored at first event of interest.

Results for all secondary outcomes stratified by sex, age, SDI and diabetes are shown in Figs. 2a,b. The number of mortality in the Nirmatrelvir-Ritonavir, Molnupiravir and control groups, stratified by organ dysfunction are shown in Table 3; The number of organ dysfunctions and length of stay, stratified by survival status are shown in Supplementary Table 3.

**Table 3:** Number of mortalities in the nirmatrelvir-ritonavir, molnupiravir and control groups, stratified by organ dysfunction.

	Number of mortalities			*P* value
	Nirmatrelvir-ritonavir	Molnupiravir	Control	
Circulatory shock	38 (60.3)	378 (54.4)	7 (63.6)	0.561
Respiratory failure	226 (54.7)	2233 (42.0)	50 (48.5)	< 0.001
Acute kidney injury	81 (100.0)	518 (98.7)	21 (100.0)	0.503
Coagulopathy	19 (67.9)	190 (91.3)	10 (83.3)	0.001
Acute liver impairment	5 (83.3)	88 (100.0)	4 (100.0)	< 0.001
Organ dysfunction composite	293 (57.6)	2577 (44.0)	79 (57.7)	< 0.001

## Limitations

Hong Kong has a robust and comprehensive database from which we acquired real-world clinical data for analysis. However, patients’ symptomatology, SARS-CoV-2 vaccination status and vital sign measurements throughout the health care process were not available. Moreover, medical compliance data was not available, and therefore fidelity for these confounders cannot be adjusted.

With the recent availability of these antivirals and the proximity to the outbreak, this study has short follow-up, meaning that late outcomes/effects, for example, prevention of long-term consequences of infection or associated health service utilization, are not assessable during the study period.

## Discussion

This analysis from the real-life data among patients infected with the Omicron variant in Hong Kong investigated the association of Molnupiravir and Nirmatrelvir-Ritonavir with reduced mortality and sepsis. In this study, both antivirals were associated with large degrees of reduction in all-cause and respiratory mortalities, which is compatible with published literature^11,35–37^, indicating its potential contribution to contain the infection from the epidemic.

Sepsis is a clinical syndrome that has physiologic and biochemical abnormalities caused by a dysregulated host response to infection. Sepsis and the inflammatory response that ensues can lead to multiple organ dysfunction syndrome and death^12^. In a viral infection neither blood nor lower respiratory tract specimen culture are positive. In COVID-19, the proportion of viral sepsis among all-cause sepsis was reported as 76%^38^. In this study, both Nirmatrelvir-Ritonavir and Molnupiravir were associated with a reduction in organ dysfunction driven by a decrease in circulatory shock and respiratory failure among high-risk hospitalized patients infected with Omicron variant.

SARS-CoV-2 was detected in the highly-vascularized heart, respiratory tract, kidneys, liver, and brain^39^. Endothelial cells express the angiotensin-converting enzyme 2 (ACE2) receptor and the cellular proteases, which are favorable to SARS-CoV-2 and subsequent virus dissemination to various organs through vasculature. A very high level of cytokine facilitates the development of cytokine storm in COVID-19^40^.

Influenza viruses, including influenza A and B, can cause both seasonal epidemics and out-of-season sporadic cases and outbreaks^41^. A previous report from the USA found that the incidence rate of influenza-associated sepsis hospitalization was 8.8/100,000 person-years (95% CI 3.9–16.5)^42^. The relative risk of sepsis and septic shock in 2009 pandemic influenza A was 1.70 (95% CI 1.46–1.97) over seasonal influenza^43^. A report from Wuhan suggesting that COVID-19 (wild-type) associated sepsis and septic shock can be found in 59% and 20% of patients respectively. However, at the time of writing, the data on Omicron variant-associated sepsis and septic shock was not available. In this study. the incidence of sepsis and septic shock among hospitalized patients with Omicron variant was 42.8% (N = 7593) and 5.8% (N = 1029) respectively. Even though the figure is lower than wild-type, an infection with Omicron may pose a huge burden to health care systems due to its highly transmissible nature.

In this study, the effect of Nirmatrelvir-Ritonavir and Molnupiravir on acute kidney injury, acute liver impairment and coagulopathy were not significant. We reproduced the results with bootstrapping methods to obtain precise estimates of incidence rate ratios, hazard ratios and confidence intervals. After bootstrapping, the antivirals were significantly additionally associated with reduced acute liver impairment, suggesting its potential effect on liver or kidney dysfunctions.

The effectiveness and utility of Nirmatrelvir-Ritonavir and Molnupiravir have been evaluated in other observational studies^44–47^. One Italian study similarly found decreased risk of death and hospitalization associated with COVID-19 antiviral use^44^. In a study of hospitalized patients without need of supplemental oxygen, use of Nirmatrelvir-Ritonavir (HR: 0.34 [95% CI 0.23–0.50]) and Molnupiravir (HR 0.48 [95% CI 0.40–0.59]) decreased all-cause mortality^45^. In another study conducted on non-hospitalized, community-dwelling patients, Nirmatrelvir-Ritonavir decreased risk of hospitalization (HR 0.76 [95% CI 0.67–0.86]) but not Molnupiravir (HR 0.98 [95% CI 0.89–1.06])^46^. In terms of cost-effectiveness, both Nirmatrelvir-Ritonavir and Molnupiravir were associated with healthcare system cost savings^47^.

These findings should be interpreted together with vaccination status in the population. In Hong Kong, these antivirals were prescribed for individuals who have not completed vaccination against COVID-19 (either attenuated virus vaccine or messenger Ribonucleic Acid vaccine). The elderly population is largely unvaccinated and more likely to develop severe COVID-19. As of April 15, 2022, only 40.0% of population aged 60 years or above have completed vaccination (3 doses), of which 60 to 69 years 50.0%, 70–79 years 38.4% and 80 years or above 14.9%^48^. These observations warrant further analysis, including the number of vaccine doses, to explore the underlying factors accounting for differences in antiviral efficacy among different age groups.

In subgroup analysis, it was found that antivirals had no significant reduction in outcomes among patients aged 60 or under, in contrary to patients from other age groups. While further analysis is warranted for the underlying cause, these patients have additional risk factors for severe disease, suggesting comorbidities and disease factors, rather than age, contribute more to all-cause sepsis.

In summary, this analysis of real-world application of oral antivirals to patients infected with Omicron variant demonstrated that both Molnupiravir and Nirmatrelvir-Ritonavir were associated with a reduction in all-cause and respiratory mortality, and sepsis. Further research, including from randomized clinical trials, is needed to more definitively determine the role of antiviral therapy in high-risk COVID-19 adults for primary prevention of all-cause mortality and sepsis.

## Supplementary Information

Supplementary Information 1.

Supplementary Information 2.

Supplementary Information 3.

Supplementary Information 4.

## Data Availability

The datasets generated and analyzed during the current study are available from the corresponding author on reasonable request.

## Abbreviations

ACE: Angiotensin-converting enzyme
AKI: Acute kidney injury
ASE: Adult sepsis event
CCI: Charlson comorbidity index
CDARS: Clinical data analysis and reporting system
CDC: Centers for disease control and prevention
HA: Hospital authority
HR: Hazard ratio
ICD-9-CM: International classification of diseases, ninth revision, clinical modified
IPTW: Inverse probability treatment weighting
IRD: Incidence rate difference
RdRp: RNA-dependent RNA polymerase
SD: Standard deviation
SDI: Social deprivation index
SMD: Standardized mean difference
VOC: Variant of concern
