# Supplementary material for: Association of Molnupiravir and Nirmatrelvir-Ritonavir with reduced mortality and sepsis in hospitalized omicron patients: a territory-wide study
Source: Sci Rep. 2023 May 15;13:7832. doi: 10.1038/s41598-023-35068-w (PMC10183691; doi:10.1038/s41598-023-35068-w)
Supplement: Supplementary file 1 — Supplementary Information 1. [file 41598_2023_35068_MOESM1_ESM.docx]

**Supplementary Table 1: Definition of Study Outcomes**

| **Primary outcomes** |  |
| --- | --- |
| Respiratory mortality | ICD-10 code J00-J99, and U071 |
| **Secondary outcomes** |  |
| Circulatory shock | Initiation of a vasopressor or inotrope |
| Respiratory failure | Initiation of invasive mechanical ventilation (identified by ICD-9 procedure codes 93.90, 93.91, 96.70, 96.71, 96.72) OR initiation of dexamethasone |
| Acute kidney injury | Doubling of serum creatinine from the lowest value during hospitalization OR decrease by ≥50% of estimated glomerular filtration rate relative to highest value during hospitalization. |
| Coagulopathy | Platelet count <100 cells/µL AND > 50% decline from highest value during hospitalization. |
| Acute liver impairment | Total bilirubin ≥2.0mg/dL AND increase by 100% from lowest value during hospitalization. |
| Organ dysfunction | Presence of any one of the above secondary outcomes |

**Supplementary Table 2: International Classification of Diseases, Ninth Revision, Clinical-Modification (ICD9-CM) Diagnosis Codes for Comorbidities**

| **Condition** | **ICD-9 codes** |
| --- | --- |
| Diabetes | 250.00-250.99 |
| Hypertension | 401.00-405.99 |
| Stroke | 362.30-362.49; 430.00-435.99 |
| Heart failure | 428.00-428.99 |
| Atrial fibrillation | 427.31 |
| Parkinson’s’ disease | 332.00 |
| Schizophrenia | 295.00-295.99 |
| Liver cirrhosis | 571.50-571.59, 571.2, 546.0, 456.00-456.21, 567.00-567.21, 567.89-567.99, 572.20-572.49, 789.59 |
| Depression | 269.20-269.39; 269.50-269.59; 300.4; 309.00-311.00 |
| Kidney disease | 583.00-586.99; 592.00-592.99; 593.9 |
| Rheumatoid arthritis | 714.00-714.29; 446.5; 714.80-718.89; 725.0 |
| Obesity | 277.70-280.09 |
| Alcohol abuse | 303.00-303.99; 305.0 |

**Supplementary Table 3: Number of organ dysfunctions and length of stay, stratified by survival status**

|  | Survivors  (n=15008) | Non-survivors  (n=2696) | P value |
| --- | --- | --- | --- |
| *Circulatory Shock* | 403 (2.7) | 366 (13.6) | <0.001 |
| *Respiratory Failure* | 4239 (28.2) | 1590 (59.0) | <0.001 |
| *Acute Kidney Injury* | 590 (3.9) | 37 (1.4) | <0.001 |
| *Coagulopathy* | 204 (1.4) | 44 (1.6) | 0.307 |
| *Acute Liver Impairment* | 79 (0.5) | 19 (0.7) | 0.313 |
| *Organ Dysfunction Composite* | 4779 (31.8) | 1719 (63.8) | <0.001 |
| *Length Of Stay (Days[SD])* | 7.02 (6.98) | 4.68 (5.20) | <0.001 |

**Supplementary Figure 1: Bootstrap method followed by sampling with replacement**

We used first bootstrapping and then sampling with replacement which applied the central limit theorem to obtain the estimates of hazard ratios, weighted incidence rate differences and 95% confidence intervals for every subgroup.

For a subgroup with an outcome, by bootstrapping stratified with censoring status, we created 1000 samples of the data frame, from each of which a weighted incidence rate difference (weighted IRD) and a hazard ratio (HR) were computed.

However, the distributions where the weighted IRD and HR followed were unknown, which makes the derivation of 95% confidence intervals difficult. Rather than directly taking sample means as estimates of weighed IRD and HR, those 1000 HRs and weighted IRDs from bootstrapped samples would represent a population of HR and a population of weighed IRD.

Then via sampling with replacement for 100 times in each population, we obtained a sample of 100 HRs and a sample of 100 weighed IRDs then obtained a sample mean of HRs and a sample mean of weighted IRDs. Then the process of obtaining 100 HRs and 100 weighted IRDs was repeated 2000 times. According to the central limit theorem, which states that sample means from independent and identically distributed random variables will tend to follow a normal distribution while the number of data to compute the sample mean increases, the sample means of weighted IRD and HR were also in normal distributions, where the standard errors were estimated from these 2000 sample means of weighted IRDs and HRs. The ultimate estimates of HR and weighted IRD, shown in the table in this paper, were sample means of those 2000 means of HR and weighted IRD.

The 95% confidence intervals for hazard ratio and weighted incidence rate difference for a subgroup and an outcome were shown as follows:

95% confidence interval for a hazard ratio:

$$\left[ \overline{\overline{X_{hr}}}\pm1.96\times\sigma_{\overline{X_{hr}}} \right]$$

And thus the 95% confidence interval for a weighted incidence rate difference:

$$\left[ \overline{\overline{X_{IRD}}}\pm1.96\times\sigma_{\overline{X_{IRD}}} \right]$$

**Supplementary Figure 2: Study Flow Chart**

**
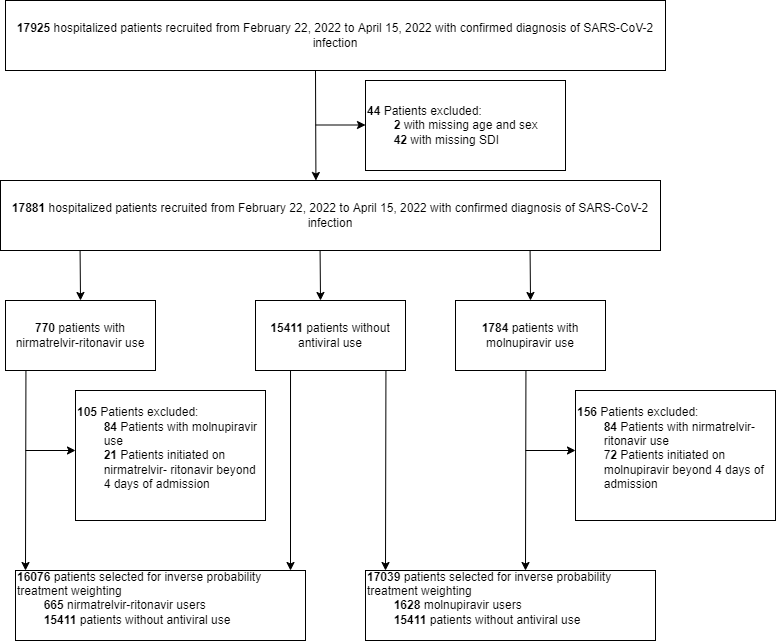
**
