# Supplementary material for: Association of Molnupiravir and Nirmatrelvir-Ritonavir with reduced mortality and sepsis in hospitalized omicron patients: a territory-wide study
Source: Sci Rep. 2023 May 15;13:7832. doi: 10.1038/s41598-023-35068-w (PMC10183691; doi:10.1038/s41598-023-35068-w)
Supplement: Supplementary file 2 — Supplementary Information 2. [file 41598_2023_35068_MOESM2_ESM.docx]

**Supplementary Figure 3: Association of Antivirals Use with Mortality and Organ Dysfunction after Bootstrapping**

Legend: (a) Association between Nirmatrelvir-Ritonavir and Molnupiravir Use, All-Cause Mortality, and Major Organ Dysfunction Events after Bootstrapping, in 17.704 Hospitalized Patients Infected with Omicron Variant of SARS-CoV-2; (b) Association between Nirmatrelvir-Ritonavir and Molnupiravir Use, All-Cause and Respiratory Mortality after Bootstrapping, Stratified by Age, Sex, SDI and Diabetes.

Abbreviations: IRD: Incidence Rate Difference; HR: Hazard Ratio; SDI: Social Deprivation Index.

Weighted incidence rate differences comparing Nirmatrelvir-Ritonavir and Molnupiravir Users to non-users after inverse probability weighting was applied.
Composite: Time to first circulatory shock, respiratory failure, coagulopathy, acute kidney injury and acute liver impairment. There were fewer composite events compared with total individual events, as participants were censored at first event of interest.

**Supplementary Figure 4: Association of Antivirals Use with Organ Dysfunction after Bootstrapping, Stratified by Age, Sex, SDI and Diabetes**

Legend: (a) Association between Nirmatrelvir-Ritonavir and Major Organ Dysfunction Events after Bootstrapping, in Hospitalized Patients Infected with Omicron Variant of SARS-CoV-2; (b) Association between Molnupiravir and Major Organ Dysfunction Events after Bootstrapping, in Hospitalized Patients Infected with Omicron Variant of SARS-CoV-2.

Abbreviations: IRD: Incidence Rate Difference; HR: Hazard Ratio; SDI: Social Deprivation Index.

Weighted incidence rate differences comparing Nirmatrelvir-Ritonavir and Molnupiravir Users to non-users after inverse probability weighting was applied.
Composite: Time to first circulatory shock, respiratory failure, coagulopathy, acute kidney injury and acute liver impairment. There were fewer composite events compared with total individual events, as participants were censored at first event of interest.
