# Supplementary figures and images for: Association of Molnupiravir and Nirmatrelvir-Ritonavir with reduced mortality and sepsis in hospitalized omicron patients: a territory-wide study
Source: Sci Rep. 2023 May 15;13:7832. doi: 10.1038/s41598-023-35068-w (PMC10183691; doi:10.1038/s41598-023-35068-w)

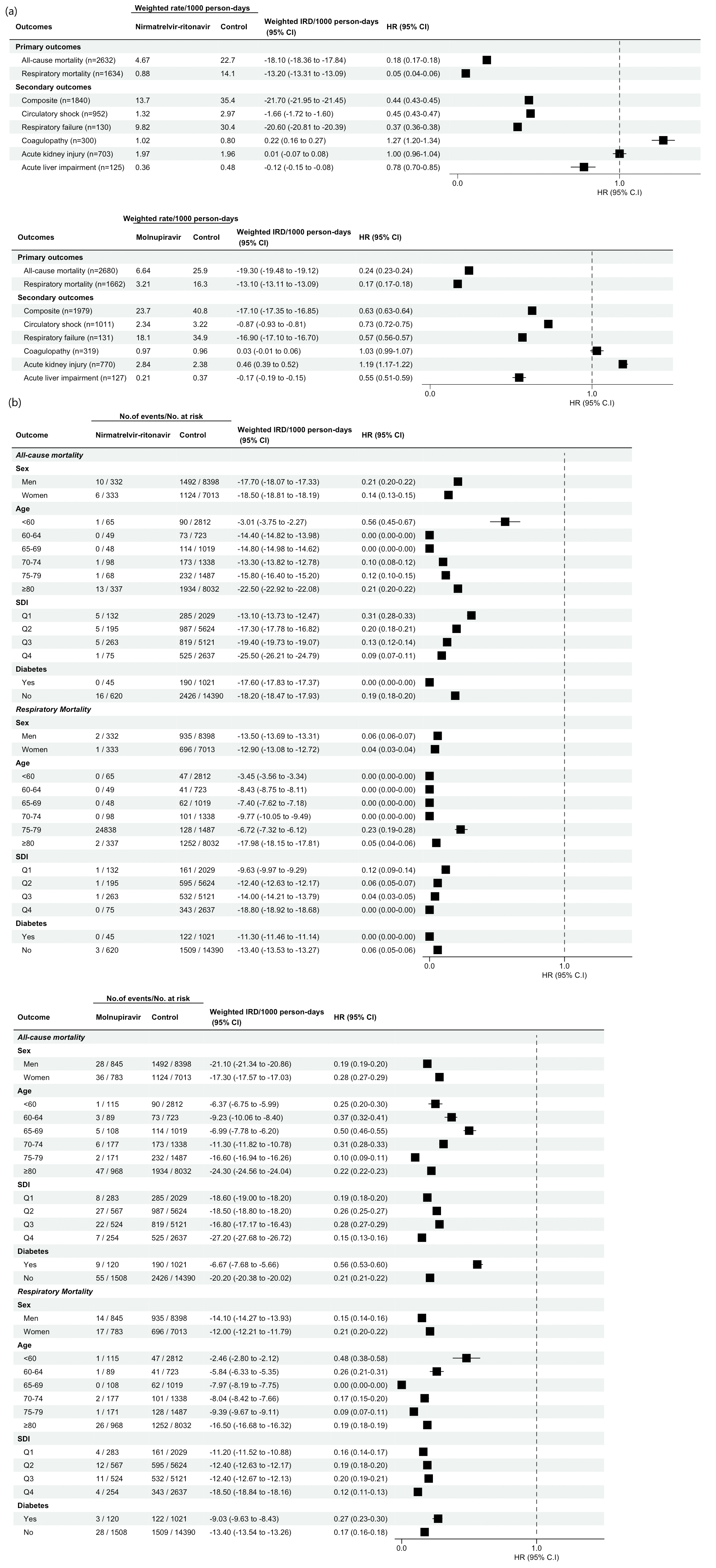

Supplement: Supplementary file 3 — Supplementary Information 3. [file 41598_2023_35068_MOESM3_ESM.png]

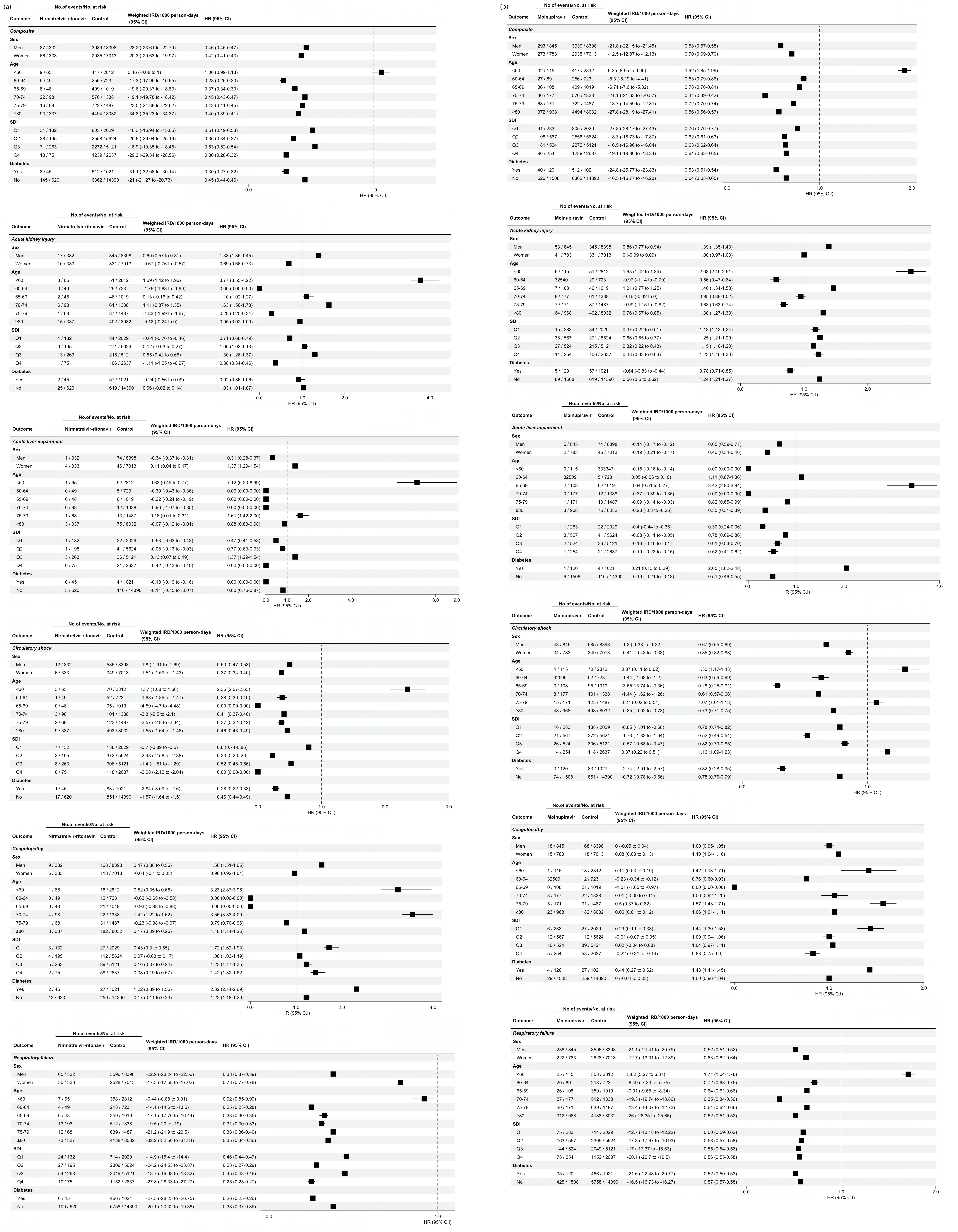

Supplement: Supplementary file 4 — Supplementary Information 4. [file 41598_2023_35068_MOESM4_ESM.png]
